# Does directly observed therapy (DOT) reduce drug resistant tuberculosis?

**DOI:** 10.1186/1471-2458-11-19

**Published:** 2011-01-07

**Authors:** Patrick K Moonan, Teresa N Quitugua, Janice M Pogoda, Gary Woo, Gerry Drewyer, Behzad Sahbazian, Denise Dunbar, Kenneth C Jost, Charles Wallace, Stephen E Weis

**Affiliations:** 1 University of North Texas Health Science Center at Fort Worth, Department of Medicine, Fort Worth, TX, USA; 2 Tarrant County Health Department, Fort Worth, TX, USA; 3 University of Texas Health Science Center at San Antonio, Department of Microbiology and Immunology, San Antonio, TX, USA; 4 Statology, Ventura, CA, USA; 5 Dallas County Health And Human Services, Dallas, TX, USA; 6 Texas Department of State Health Services, Austin, TX, USA; 7 Centers for Disease Control and Prevention, Atlanta, GA, USA

## Abstract

**Background:**

Directly observed therapy (DOT) is a widely recommended and promoted strategy to manage tuberculosis (TB), however, there is still disagreement about the role of DOT in TB control and the impact it has on reducing the acquisition and transmission of drug resistant TB. This study compares the portion of drug resistant genotype clusters, representing recent transmission, within and between communities implementing programs differing only in their directly observed therapy (DOT) practices.

**Methods:**

Genotype clusters were defined as 2 or more patient members with matching IS*6110 *restriction fragment length polymorphism (RFLP) and spoligotype patterns from all culture-positive tuberculosis cases diagnosed between January 1, 1995 and December 31, 2001. Logistic regression was used to compute maximum-likelihood estimates of odds ratios (ORs) and 95% confidence intervals (CIs) comparing cluster members with and without drug resistant isolates. In the universal DOT county, all patients received doses under direct observation of health department staff; whereas in selective DOT county, the majority of received patients doses under direct observation of health department staff, while some were able to self-administer doses.

**Results:**

Isolates from 1,706 persons collected during 1,721 episodes of tuberculosis were genotyped. Cluster members from the selective DOT county were more than twice as likely than cluster members from the universal DOT county to have at least one isolate resistant to isoniazid, rifampin, and/or ethambutol (OR = 2.3, 95% CI: 1.7, 3.1). Selective DOT county isolates were nearly 5 times more likely than universal DOT county isolates to belong to clusters with at least 2 resistant isolates having identical resistance patterns (OR = 4.7, 95% CI: 2.9, 7.6).

**Conclusions:**

Universal DOT for tuberculosis is associated with a decrease in the acquisition and transmission of resistant tuberculosis.

## Background

Worldwide tuberculosis transmission continues despite intensive control efforts and availability of highly effective, relatively inexpensive treatment regimens [1-3]. There are multiple reasons for the continued threat. The time commitment for successful treatment of tuberculosis is longer than required for most acute medical conditions [4,5]. Additional tuberculosis transmission and the development of drug resistance can result when tuberculosis treatment fails due to noncompliance [6]. Treatment noncompliance is difficult to predict and identify prior to treatment failure [7-13]. A widely recommended and promoted strategy attempting to manage the situations leading to noncompliance is commonly referred to as directly observed therapy (DOT) [14-16]. Nonetheless, there is still disagreement about the role of DOT in tuberculosis control [17-19].

The skepticism surrounding DOT results from a lack of a compelling scientific rationale supporting its effectiveness. The rationale for DOT is based on treatment outcomes in adherent patients in research settings; clinical experiences of treatment failure related to non-compliance; and DOT studies using a variety of designs, methodologies, and definitions [1-5,18,20-22]. There are practical impediments to completing well-designed studies on DOT efficacy; perhaps chief among these is an ethical issue. An essential component of the protection of human subjects in clinical trials is subjects' ability to withdraw from a study at any time with no loss of privileges or rights [23]. Unlike most illnesses evaluated through randomized trials, tuberculosis is a public health problem that affects others in the community through respiratory transmission. As a result, laboratories and physicians in some areas are legally mandated to report the identity of persons with tuberculosis to health authorities [24]. To prevent tuberculosis transmission, persons with tuberculosis who are treatment-noncompliant can face loss of personal liberty and completion of therapy can be a condition to regain liberty [24-26]. Randomization to a treatment strategy where study therapy, if not taken, can result in treatment being legally mandated by public health authorities is problematic [23]. This lack of randomized trials has resulted in a lack of consensus on the program components that are essential for a tuberculosis treatment policy to be defined as DOT. As a result, studies of DOT have included different combinations of incentives and enablers, tracing of defaulters, legal sanctions, patient-centered approaches, staff motivation, supervision, and funding [18,20-22]. Thus the efficacy of DOT remains controversial at the same time it is widely recommended [14,17-19].

The DOT policy for tuberculosis patients in Texas is a comprehensive program that includes medical services related to TB and delivery of medication to a patient-chosen location at no cost to the patient. For patients who default or decline therapy there is a legal framework and dedicated facilities for inpatient hospital isolation, (i.e., quarantine), until therapy is completed. The two counties compared in this study are located in urban north central Texas and had an incidence of 10.4 to 8.2 cases per 100,000 population (County A 1995, 2001) and 8.8 to 6.5 cases per 100,000 population (County B 1995, 2001). The tuberculosis control programs of the two counties contain the same elements. Both programs are managed by the local county health departments, funded through the same formula to provide services to tuberculosis patients, under the same contract standards, and overseen by the Texas Department of State Health Services. Full microbiological laboratory services, including susceptibility testing, are equally available for each patient and use the same standards. First- and second-line anti-tubercular medications are provided from the same central state-managed pharmacy. Treatment decisions in both counties are made by clinicians who follow Centers for Disease Control and Prevention (CDC)/American Thoracic Society treatment guidelines and have also participated in CDC sponsored research in tuberculosis control. The principal management difference between these counties is that they utilized different DOT strategies prior to and during the time of this study. One county had provided DOT to the majority of patients since 1986 while the remainder received traditional self-administered therapy (selective DOT); this is standard practice in most of the US. There were no rigid criteria for managing a patient with DOT. General criteria for DOT management included: clinician judgment that the patient would benefit from DOT, a prolonged period of smear or culture positivity, and irregular attendance at scheduled visits.

The other county provided DOT to all persons suspected of having or proven to have tuberculosis within the county (universal DOT) during the same time period. If DOT is not more effective for managing tuberculosis than is self-supervision of therapy, then acquisition and transmission of drug resistant strains should be similar in both counties. A molecular epidemiologic analysis of tuberculosis isolates representing transmission and drug susceptibilities is reported here.

## Methods

As part of their participation in the National Tuberculosis Genotyping and Surveillance Network sponsored by CDC [27,28], the Texas Department of State Health Services and the University of North Texas Health Science Center at Fort Worth conducted a community-based cohort study including two urban north Texas counties. The University of Texas Health Science Center at San Antonio (UTHSC-SA) laboratory served as the reference genotyping laboratory for *M. tuberculosis *complex isolates collected from each tuberculosis patient residing in the counties from January 1, 1995 to December 31, 2001. The IS*6110 *restriction fragment length polymorphism (RFLP) fingerprint patterns of isolates were submitted to the CDC and assigned a National Fingerprint Pattern [28].

### Drug susceptibility testing

Drug susceptibility testing of the *M. tuberculosis *complex isolates was performed with the rapid BACTEC 460TB radiometric system (Becton Dickinson, Paramus, N.J.). The anti-tuberculosis drugs and concentrations tested by the BACTEC 460TB system were as follows: isoniazid (INH), 0.4 mcg/ml; rifampin (RIF), 2.0 mcg/ml; and ethambutol (EMB), 2.5 mcg/ml [29]. Resistance to any of the anti-tuberculosis agents was confirmed by the Middlebrook 7H10 agar proportion method. The anti-tuberculosis drugs and concentrations tested by the agar proportion method were as follows: INH, 1.0 mcg/ml; RIF, 1.0 mcg/ml; and EMB, 5.0 mcg/ml. Drug susceptibilities reported were those obtained from the initial isolate available and represent initial resistance. Initial resistance in this study is a combination of primary resistance in persons without prior treatment and acquired resistance in persons who have been previously treated.

### Cluster definitions

Molecular characterization of *M. tuberculosis*, can help elucidate transmission dynamics in communities. IS*6110-*RFLP and spoligotype analyses were conducted on all *M. tuberculosis *isolates as previously described [30]. Genotype clusters are thought to represent recent transmission [27-30]. Clusters were defined as groups of two or more isolates having an identical spoligotype and IS*6110 *RFLP pattern, allowing for the presence, absence or shifting of no more than one band. Because we were interested in both initial drug resistance and the direct transmission of a drug-resistant strain, subsequent genotype clusters were further categorized as: (a) having no drug resistant isolates from any cluster members, (b) "resistant clusters" having at least one isolate resistant to INH, RIF, or EMB from any cluster members; or (c) "two-isolate resistant clusters" having at least two members with isolates resistant to INH, RIF or EMB.

### Data analysis

Texas State statute requires laboratories to report to the health authority persons with cultures positive for *M. tuberculosis*. Similarly clinicians are required to report persons suspected or known to have tuberculosis. Data from local health department tuberculosis databases from each county was merged with a database of IS*6110 *RFLP fingerprint patterns from UTHSC-SA laboratory. The merge was done by name, date of birth, and tuberculosis episode number. To maximize the number of one-to-one matches between the databases, extensive efforts were made to correct inconsistencies and inaccuracies in both databases, such as name misspellings and incorrect dates of birth. Isolates that could not be matched were excluded from the study.

Demographic and clinical data was obtained for local Health Department records. Due to incomplete coding, a substantial proportion of records had missing foreign-born (8%) and/or HIV status (13%). Original medical records of a sample of subjects with missing data were referenced to determine whether or not these data were actually missing or left uncoded; based on this process, it was determined that uncoded foreign-born and HIV status variables had actual values of "no" and were therefore coded as such in the merged database. Treatment defaulters were defined as patients who were reported as being lost.

Differences in age were tested using t-tests. All other variables were categorical and were tested using Fisher's exact tests or chi square goodness-of-fit tests for comparisons to population distributions. Logistic regression was used to compute maximum-likelihood estimates of odds ratios (ORs) and 95% confidence intervals (CIs), using both uni- and multivariable models. All isolates were treated as independent units of analysis, although a small percentage of patients (0.8%) had multiple isolates in the database due to recurrent tuberculosis episodes separated by at least one year and occurring between 1995 and 2001. All tests were two-sided with an alpha of 0.05.

Treatment outcome data and the method of therapeutic treatment (e.g. directly observed therapy vs. self-administered therapy) were collected as part of routinely reported variables to the CDC, U.S. National Tuberculosis Surveillance System. Comparisons of proportions for these data include all reportable cases for the study period, not just culture-positive cases with valid genotype results.

The institutional review boards of the Centers for Disease Control and Prevention, the University of North Texas Health Science Center at Fort Worth, and the UTHSC-SA have approved this investigation.

## Results

**Figure 1:**
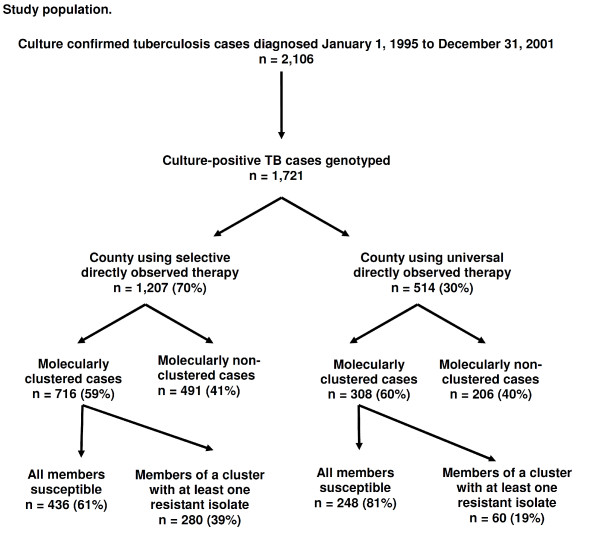
**Study population**.

From January 1, 1995 to December 31, 2001, there were 2,106 culture confirmed cases of *M. tuberculosis *in the two counties [31]. Of these, 1,721 viable clinical isolates from 1,706 (81%) patients were available to the reference genotyping laboratory for molecular analysis. Persons excluded from analysis because there was no viable clinical isolate of *M. tuberculosis *did not differ statistically from those with viable isolates by age (*P *= 0.44), gender (*P *= 0.56), or race (*P *= 0.70). One thousand twenty four (60%) patients genotyped met the criteria for molecular clustering. These patients were categorized into 148 clusters varying from 2 to 138 patients per group. Twenty-six (1.5%) of the isolates had an identical low copy RFLP pattern but did not have spoligotyping conducted and were included as non-clustered isolates (Figure 1).

Foreign birth and gender of tuberculosis patients did not vary by county(data not shown). While age distributions of the general populations of these two counties were the same (8% for those under 5 yrs, 20% for 5 - 17 yrs, 64% for 18 - 64 yrs, and 8% for those over 65 yrs.; Table 1), the mean age at diagnosis was younger in the selective DOT county; this difference was largely due to the age distribution of clustered patients (40.4 ± 15.3 years in selective DOT vs 43.4 ± 15.9 years in universal DOT among clustered patients, *P *= 0.004). Race/ethnicity distributions of the general populations of these counties were not similar, with the selective DOT county having a more racially diverse population than the universal DOT county. As shown in Table 1 in the selective DOT county disproportionally affected blacks and Asians were over-represented and non-Hispanic whites were underrepresented compared to the general population. In the universal DOT county, blacks, Hispanics, and Asians were overrepresented and whites were underrepresented. The selective DOT county had a higher percentage of tuberculosis patients with HIV co-infection than the universal DOT county (15% [n = 183] vs. 11% [n = 58], *P *= 0.03), with this difference was greatest among clustered patients (19% [n = 135] vs. 13% [n = 39], *P *< 0.0001); the prevalence of HIV in the general populations of the two counties is unknown. Forty-one percent of patients reported with tuberculosis (i.e., culture-confirmed and clinical cases) in the selective DOT county received DOT therapy versus 97% in the universal DOT county. The selective DOT county had a higher percentage of treatment defaulters (i.e., lost to follow-up) than the universal DOT county (7.9% [n = 134] vs. 2.9% [n = 22]; p < 0.001). The selective DOT county had a lower percentage of patients completing treatment than the universal DOT county (85.9% [n = 1,448] vs. 90.7% [n = 745]; p < 0.01). Recurrent TB was not significantly different between counties.

**Table 1:** Demographic characteristics by county.

		Selective DOT County			Universal DOT County		
		TB Patients	General Population	*p*-value	TB Patients	General Population	*p*-value
Demographic		(n = 1,194)			(n = 512)		
Age (years)	<5	2%	8%	<0.0001	1%	8%	<0.0001
	5 - 17	3%	20%		2%	20%	
	18 - 64	85%	64%		84%	64%	
	≥65	10%	8%		12%	8%	
Sex	Male	66%	50%	<0.0001	67%	49%	<0.0001
	Female	34%	50%		33%	51%	
Race/ethnicity	Black	42%	20%	<0.0001	29%	13%	<0.0001
	Hispanic	29%	30%		24%	20%	
	White	17%	44%		31%	62%	
	Asian	11%	4%		15%	4%	
	Other	0%	2%		1%	1%	

Table 2 shows comparisons by county on isolate characteristics. One hundred twenty (7%) of the isolates were resistant to at least INH, RIF, or EMB. Adjusting for age, race, and HIV status, antitubercular drug resistance to INH, RIF and/or EMB in the initial isolate was over 1.5 times as common in the selective than in the universal DOT county (p = 0.049). Isolates belonging to clusters containing at least one isolate resistant to INH, RIF and/or EMB ("resistant" clusters) were more than twice as likely to occur in the selective than in the universal DOT county (p < 0.0001). Isolates belonging to clusters with at least two isolates with resistant members having the identical resistance pattern ("two-isolate resistant" clusters) were over 4 times more likely in the selective than in the universal DOT county (*p *< 0.0001). When the analysis was confined to clusters with all members exclusively in either county, isolates belonging to resistant clusters remained significantly more common in the selective DOT county (OR = 3.8, 95%CI; 1.6, 8.8), as did isolates belonging to two-isolate resistant clusters (OR = 5.1, 95%CI; 1.2, 21.7) (data not shown). Frequency of relapse did not vary significantly by county during the study period (1.0 vs 0.3%, *p *= 0.26). In the selective DOT county 9 of 13 relapses were with the same strain vs 1 of 2 relapses in the universal DOT county (*p *= 1.00). Stratified by demographic factors that significantly differed by county, INH, RIF and/or EMB resistance appeared to be increasingly associated with the selective DOT county with increasing age, and seemed more associated with selective DOT among blacks, Asians, and other ethnicities compared to whites and Hispanics (Table 3). None of these potential effect modifiers reached statistical significance. HIV status was not an effect modifier.

**Table 2:** Number of isolates by isolate characteristics and type of county DOT program.

	Selective DOT county		Universal DOT county							
	(n = 1207)		(n = 514)		Crude			**Adjusted**^**1 **^		
Isolate characteristic	**No**.	(%)	**No**.	(%)	OR	(95% CI)	p-value	OR	(95% CI)	p-value
EMB/INH/RIF resistant^2^										
No	1108	(92)	489	(95)	1.0		0.03	1.0		0.049
Yes	95	(8)	25	(5)	1.7	(1.1, 2.6)		1.6	(1.1, 2.6)	
Unknown	4									
Belongs to a resistant cluster^3^										
No	927	(77)	454	(88)	1.0		<0.0001	1.0		<0.0001
Yes	280	(23)	60	(12)	2.3	(1.7, 3.1)		2.1	(1.6, 2.9)	
Belongs to a two-isolate resistant cluster^4^										
No	1024	(85)	495	(96)	1.0		<0.0001	1.0		<0.0001
Yes	183	(15)	19	(4)	4.7	(2.9, 7.6)		4.3	(2.6, 7.0)	

^1^Adjusted for age, race, and HIV status.

^2^Resistant to EMB, INH, and/or RIF.

^3^Genotype-clustered with at least one isolate in cluster resistant to EMB, INH, and/or RIF.

^4^Genotype-clustered with at least two of the EMB-, INH- and/or RIF-resistant isolates having the same EMB-INH-RIF resistance pattern.

**Table 3:** Differences in EMB/INH/RIF resistance by age, race, and HIV status, by county DOT program type1

	Selective DOT county		Universal DOT county					
	(n = 1207)		(n = 514)					Interaction
Stratifying factor	No. Resistant	(%)	No. Resistant	(%)	OR	(95% CI)	p-value	**p-value**^**2**^
Age (yrs)								0.06
<30	28	(9)	8	(7)	1.3	(0.5, 2.9)	0.58	
30 - 59	59	(8)	15	(5)	1.7	(0.9, 3.1)	0.08	
60+	8	(5)	2	(2)	2.1	(0.4, 10.2)	0.36	
Race								0.11
Black	41	(8)	6	(4)	2.1	(0.9, 5.1)	0.09	
Hispanic	20	(6)	10	(8)	0.7	(0.3, 1.5)	0.34	
White	8	(4)	3	(2)	1.4	(0.3, 5.6)	0.65	
Asian/other	26	(18)	6	(8)	2.8	(1.1, 7.1)	0.03	
HIV status								0.52
Negative	74	(7)	20	(4)	1.6	(1.0, 2.7)	0.07	
Positive	21	(11)	5	(8)	1.2	(0.4, 3.6)	0.68	

^1^Separate analyses were done for each level of each stratifying factor; e.g., among patients aged <30, 28 (9%) patients from selective DOT and 8 (7%) from universal DOT were resistant, with an OR of 1.3 associating increased resistance with selective DOT. Analyses were adjusted for age, race, and/or HIV status, as appropriate, depending on the stratification factor.

^2^Interaction between DOT program and the stratifying factor.

## Discussion

This is the first study to combine molecular genotyping techniques and drug susceptibilities to demonstrate that DOT for tuberculosis is associated with less initial drug resistance and less transmission of drug resistant organisms. Because the susceptibilities were taken from the first available isolate, the initial resistance in this study represents a combination of primary resistance and acquired resistance in patients with previous treatment. Isolates from genotype clusters containing members with initial resistance were over 1.5 times as common in the selective DOT county compared to the universal DOT county. Universal DOT was also associated with decreased transmission of drug resistant strains. Isolates from genotype clusters having 2 or more members with matching drug resistance profiles were over 4 times as common in the selective DOT county as in the universal DOT county. These findings remained significant when the cluster definition was restricted from all clusters to clusters whose members were within only one county. Since other program components in the counties are the same, this association is additional evidence that the acquisition and transmission of resistant tuberculosis is reduced by DOT.

Historically, it has been unclear whether or not DOT reduces tuberculosis transmission. It is generally accepted that most transmission of tuberculosis occurs prior to diagnosis and treatment [32,33]. DOT could reduce tuberculosis transmission by ensuring that contagious patients become noninfectious as rapidly as is possible from treatment. If DOT is more effective, then tuberculosis relapse with additional transmission could be reduced.

There is large variability in the methodology of DOT programs throughout the world, as the essential components for DOT strategy success have not been systematically established through randomized trials. The World Health Organization (WHO) defines five essential elements for Directly Observed Therapy Short-course (DOTS) strategy: political commitment to control tuberculosis; microscopy services to identify tuberculosis; uninterrupted good quality drug supplies; surveillance and monitoring systems; and use of highly efficacious regimes with direct observation of treatment for at least the initial two months [15]. These criteria are sufficiently broad that a person hospitalized for the initial 2 months of treatment with all subsequent therapy being self-administered meets the definition of DOTS strategy. Confusion can result when observation of therapy is unnecessary for using DOT strategy. This lack of standardization has resulted in studies being non-comparable.

In one previously published study, DOT was defined as therapy requiring economically impoverished ill persons without transportation to come to a clinic during working hours five times weekly for 8-12 weeks and then thrice weekly until cured [18]. This study used a maximally restrictive definition of DOT referred to by one author as "supervised swallowing" and found both self-supervised and DOT strategies to be equally ineffective [17,18]. Over 40% of patients in both the self-supervised therapy and the DOT arm failed to complete therapy [17,18]. Another study defined DOT as the daily delivery of medications to the patient at home for 2 months with subsequent self-supervised therapy to cure. This study included a penalty of loss of monetary bond if the patient defaulted on treatment. This more comprehensive DOT program achieved cure rates of 85% [34]. In much of the US, programs that strive to make tuberculosis treatment maximally accessible and include observation of therapy have been called DOT programs [35]. Essential components of these DOT programs are considered to include supplying therapy at no cost to the patient at a location of the patient's preference, combined with adherence enhancing incentives and enablers and quarantine of the noncompliant [21,27]. Some have called this a strategy of making treatment easier to take than not to take [36]. These types of programs have resulted in a sustained reduction in relapse and both initial and acquired anti-tubercular drug resistance [21,22]. In our study the components of the tuberculosis programs were the same in both counties. While the current study does not demonstrate the essential components of a DOT program, our data do demonstrate that universal DOT is associated with a significantly improved treatment outcome. Importantly, this DOT effect was independent of medication supply, incentives and enablers, laboratory services, funding, tracing and legal sanctions of defaulters, and program supervision.

There are only a few drugs and regimens of proven effectiveness in tuberculosis treatment. Drug-resistant tuberculosis is therefore a major threat to public health. If drug-resistant tuberculosis becomes widely spread, the ability to control tuberculosis through medical therapy will be seriously compromised. Not only are patients infected with strains resistant to multiple drugs less likely to be cured, but the treatment is more toxic and expensive than treatment of patients with susceptible organisms [5,37-39]. The emergence and transmission of drug-resistant *M. tuberculosis *into a population can be related to a variety of program-, health provider- and patient-related factors [38,39]. Patients' noncompliance to prescribed treatment is the most important patient-related factor contributing to the development of drug resistance [9,10,12,39]. Noncompliance cannot be reliably predicted but is considered to be reduced in programs that manage patients with DOT, thus resulting in less drug resistance [21,22]. Our study supports this concept and preventing the development and transmission of drug-resistant organisms by DOT is, at present, the best strategy for dealing with resistant tuberculosis.

Previous tuberculosis studies combining molecular genotyping and epidemiology have demonstrated that clustering represents disease due to recent transmission. In this study clustering was greater in younger patients, males, non-Hispanic blacks, patients born in the United States, and patients with HIV co-infection, all factors previously associated with clustering and recent transmission [40]. We observed significant differences in age and HIV co-infection between clustered isolates identified between the two counties; namely, among patients with clustered isolates, those in the selective DOT county were significantly younger and more likely to be HIV co-infected. A potential explanation is that DOT optimizes treatment response and results in the shortest possible period of contagion after diagnosis; therefore less tuberculosis transmission involving younger and HIV infected persons in the universal DOT county. It must be noted that differences in rates of HIV infection observed between the two counties may reflect differences in rates in the general populations, which were unknown.

The principal limitation of this study was that treatment by universal or selective DOT was not randomized or controlled and we are therefore unable to say with complete assurance that the associations identified are a result of DOT. Potential confounders include demographic and income differences between the counties which may have affected transmission (i.e., overcrowding, poverty). Successful tuberculosis control programs are multifaceted and include all measures related to aggressively identifying and treating patients with tuberculosis and latent tuberculosis infection. However, drug resistance occurring within tuberculosis chemotherapy programs is created only by monotherapy [41]. We believe that the difference in DOT strategy is the only difference between the two counties that can explain the reduced acquisition and transmission of resistant tuberculosis in the universal DOT county. While we genotyped >80% of the culture-positive cases, these non-genotyped cases are a potential source of misclassification. Moreover, as the two counties are adjacent it is conceivable that more resistant tuberculosis spread from the universal DOT county to the selective DOT county, indeed 692 patients were members of clusters that spanned both counties. However, we believe this is unlikely, as resistance associations were the same in clusters exclusive to each county as clusters that were geographically distributed across both counties.

## Conclusions

These data suggest resistance is less likely to develop and be transmitted when persons with tuberculosis are managed with universal DOT as compared to selective DOT. This provides additional support for recommending management of patients with universal DOT as the best strategy for preventing the development and transmission of drug-resistant tuberculosis. For optimal control of tuberculosis it will be necessary to define which DOT program components are essential to achieve this outcome.

## Competing interests

PM, TQ, JP, GW, GD, BS, DD, KJ, CW, and SW have no financial interests and no conflicting interests.

## Authors' contributions

PM, TQ, SW designed the study, interpreted results and drafted the article. PM, TQ, GW, BS, DD, KJ and SW collected patient and laboratory data. TQ and JP performed the data management and statistical analysis. TQ, DD and KJ conducted all laboratory testing. GW, DG, CW and SW provided program oversight and supervision. All authors read and approved the final manuscript.

## Pre-publication history

The pre-publication history for this paper can be accessed here:

http://www.biomedcentral.com/1471-2458/11/19/prepub
